# Nucleus Accumbens Deep Brain Stimulation in Patients with Substance Use Disorders and Delay Discounting

**DOI:** 10.3390/brainsci8020021

**Published:** 2018-01-27

**Authors:** Canan B. Peisker, Thomas Schüller, Jan Peters, Ben J. Wagner, Leonhard Schilbach, Ulf J. Müller, Veerle Visser-Vandewalle, Jens Kuhn

**Affiliations:** 1 Department of Psychiatry and Psychotherapy, Medical Faculty, University of Cologne, Kerpener Straße 62, 50937 Cologne, Germany; thomas.schueller@uk-koeln.de (T.S.); jens.kuhn@evkln.de (J.K.); 2 Department of Psychology, Biological Psychology, University of Cologne, Bernhard-Feilchenfeld-Straße 11, 50969 Cologne, Germany; jan.peters@uni-koeln.de (J.P.), ben.jonathan.wagner@uni-koeln.de (B.J.W.); 3 Max Planck Institute of Psychiatry, Kraepelinstraße 2-10, 80804 Munich, Germany; leonhard_schilbach@psych.mpg.de; 4 Department of Psychiatry, Otto-von-Guericke-University of Magdeburg, Universitätsplatz 2, 39106 Magdeburg, Germany; ulfmueller@gmail.com; 5 Pychosomatic Hospital Buching, Rauhenbichl, 87642 Halblech, Germany; 6 Department of Stereotactic and Functional Neurosurgery, Medical Faculty, University of Cologne, Kerpener Straße 62, 50937 Cologne, Germany; veerle.visser-vandewalle@uk-koeln.de; 7 Department of Psychiatry, Psychotherapy and Psychosomatic, Johanniter Hospital Oberhausen, Steinbrinkstraße 96a, 46145 Oberhausen, Germany

**Keywords:** delay discounting, substance use disorder, opioid use disorder, alcohol use disorder, deep brain stimulation, nucleus accumbens, self-control

## Abstract

Deep brain stimulation (DBS) of the nucleus accumbens (NAc) shows first promising results in patients with severe substance use disorder (SUD), a patient group known to have deficits in self-control. One facet of self-control is the ability to forego smaller sooner rewards in favor of larger later rewards (delay discounting, DD). The NAc has been suggested to integrate motivational information to guide behavior while the consequences of NAc-DBS on DD are unknown. To this end, nine patients with SUD performed a DD task with DBS on and after a 24 h DBS off period. Furthermore, 18 healthy controls were measured to assess possible alterations in DD in patients with SUD. Our findings implicate that DD was not significantly modulated by NAc-DBS and also that patients with SUD did not differ from healthy controls. While null results must be interpreted with caution, the commonly observed association of impaired DD in SUD might suggest a long-term effect of NAc-DBS that was not sufficiently modulated by a 24 h DBS off period.

## 1. Introduction

Substance use is one of the most relevant public health problems with a lifetime prevalence of about 8% for illicit drugs [1] and 13% for alcohol [2]. Pharmacological, psychological and social therapeutic approaches are used to treat addiction with the aim of long-term abstinence, but relapse rates are high with around 40–60% [3]. The concept of self-control is closely associated with addictive behavior and is described as the capacity to evaluate information and react flexibly regarding a long-term goal under varying environmental factors [4,5]. Patients with substance use disorder (SUD) often lack self-control and ignore negative long-term consequences in favor of the immediate transient effects of the substance [6]. Accordingly, lack of self-control in regard to the drug is an important factor contributing to relapse [7].

Behavioral economics have fundamentally influenced the understanding of drug abuse by introducing the concept of delay discounting (DD), i.e., the devaluation of rewards as a function of delay [8]. Steep discounting is considered an index of diminished self-control and might partly explain why patients with SUD choose the transient effects of the drug despite negative long-term consequences, including deterioration of health, financial and social problems [8,9,10]. Patients addicted to opioids [11,12], alcohol [13,14], nicotine [9,15], cocaine [16,17] and methamphetamines [18,19] favor smaller immediate rewards over larger delayed rewards compared to healthy subjects [20] (i.e., these patients show steeper DD). Furthermore, the degree of discounting scales with severity of drug abuse [9], number of currently used substances [16] and poor treatment outcomes [21,22,23]. There is an on-going debate as to whether steeper discount rates predispose addiction or if chronic drug exposure increases discounting [15,24,25]. Bickel, Odum and Madden [15] suggest that both factors have a meaningful influence on discounting rates.

Addiction has been increasingly recognized as a chronic disease of the brain. In line with this reasoning alterations in key structures of the performance monitoring system have been reported in patients with SUD, including the nucleus accumbens (NAc) [26,27]. The NAc has been recognized as an important hub to integrate motivational information to subsequently guide behavior [28,29]. Furthermore, lesions of the NAc have repeatedly shown to impair DD in rodents [30,31,32,33,34] and increased activity in the NAc has been linked to the choice of immediate rewards in humans [35]. 

Recently, deep brain stimulation (DBS) is being discussed as an experimental treatment option for otherwise treatment refractory patients with SUD. DBS is a neuro-modulative procedure of the central nervous system based on the implantation of electrodes within subcortical structures and is routinely used to treat movement disorders like Parkinson’s disease or tremor [36]. In addition, DBS is employed in obsessive-compulsive disorder [37], Alzheimer dementia [38], Tourette syndrome [39] and SUD [40,41,42]. The application of DBS in SUD is based on the incidental finding of a patient with severe anxiety and depression treated with NAc-DBS. Although the symptoms he was initially treated for did not improve, DBS alleviated his comorbid alcohol dependency [40]. Subsequently, patients with therapy refractory SUD who received NAc-DBS show somewhat promising results [43,44,45,46] and NAc-DBS in rodent models of SUD is able to reduce drug-related behaviors [47,48,49,50,51,52]. While early accounts on the mechanisms of high-frequency DBS hypothesized a lesion effect, more recent accounts postulate an alteration of synaptic transmission and modulation of aberrant network activity [53,54]. In animals, stimulation of the NAc shell but not the core has increased impulsive behavior [55,56]. Furthermore, a recent study suggested baseline dependent effects of NAc core-DBS on DD [57].

In the current study, we aimed to test the effect of 24 h washout after several months of continuous high-frequency NAc-DBS in patients with SUD (DBS on vs. DBS off) on discount rates compared to healthy controls (session #1 vs. session #2). We predicted that after a 24 h washout period discounting would be increased (i.e., patients show more impulsiv behavior). In addition, we predicted increased discount rates for patients with SUD compared to healthy controls.

## 2. Materials and Methods

### 2.1. Participants

**Figure 1:**
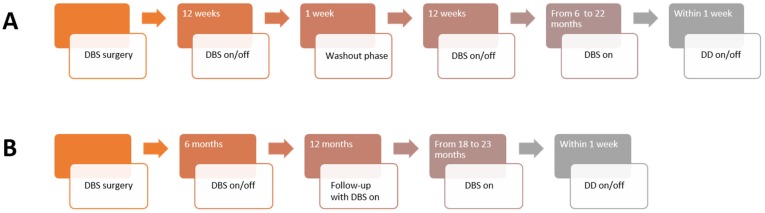
Timeline of the clinical studies and the successive delay discounting (DD) assessment. The sequence of deep brain simulation (DBS) on and off was pseudo-randomized. (**A**) The timeline of the NASA study and successive DD assessment. (**B**) The timeline of the DeBraSTRA study and successive DD assessment. (DBS, deep brain stimulation; DD, delay discounting).

Nine treatment refractory patients with SUD and NAc-DBS were enrolled in the present investigation. Five patients had been diagnosed with opioid use disorder and four patients with alcohol use disorder (mean, 42.6 years of age; standard deviation (SD), 11.3 years of age, one female, see Table 1). In addition, eighteen age and gender matched healthy controls were recruited (age 26 to 63, seven female subjects, see Table 2). All subjects provided written informed consent and the Ethics committee of the University of Cologne approved the study. The present investigation took place after the respective clinical studies. The results of the clinical studies have not been published yet. Patients with SUD performed a DD paradigm with at least 6 months of NAc-DBS on (Ø 13.3 months) and DBS off in a counterbalanced order (see Figure 1). The wash out period (DBS off) lasted at least 24 h. Healthy controls performed the DD paradigm on two different days within a week. 

### 2.2. Clinical Trials

#### 2.2.1. NASA

Patients with opioid use disorder were enrolled in the double-blind randomized crossover NASA study (CIV-1-05-0003070). The objective of the study was to assess the efficacy and safety of NAc-DBS for the treatment of opioid use disorder. The primary outcome parameters were craving and Levomethadone dosage. We hypothesized a decrease in both parameters for acute NAc-DBS. The main inclusion criteria were: (1) Heroin dependency according to DSM-IV. (2) Participation in a substitution program for at least three month. (3) A minimum of one inpatient detoxification. (4) A minimum of one long-term treatment of drug dependence. (5) Age of 18 years and older. (6) German as native language. Throughout the course of the study, there were no major complications regarding the surgical procedure or side effects of the stimulation.

#### 2.2.2. DeBraSTRA

Patients with alcohol use disorder were enrolled in the multi-centered double-blind randomized controlled DeBraSTRA study (CIV-11-05-000663). The objective of the study was to assess the efficacy and safety of NAc-DBS for the treatment of alcohol use disorder. Inclusion criteria were: (1) Alcohol use disorder for at least ten years. (2) Treatment resistance as defined by unsuccessful long-term inpatient rehabilitation of at least six months, or two or more rehabilitation treatments discontinued by the patient, and at least one pharmacological therapy for at least two months with an anti-craving drug. (3) A minimum of 30 drinks per average per week. (4) At least one inpatient detoxification. (5) No withdrawal symptoms prior to surgery. (6) Age between 25 and 60 years. (7) At least nine years of education. (8) A phone number for interviews. (9) Male gender. There were no major complications regarding the surgical procedure or side effects of the stimulation.

#### 2.2.3. Deep Brain Stimulation

**Figure 2:**
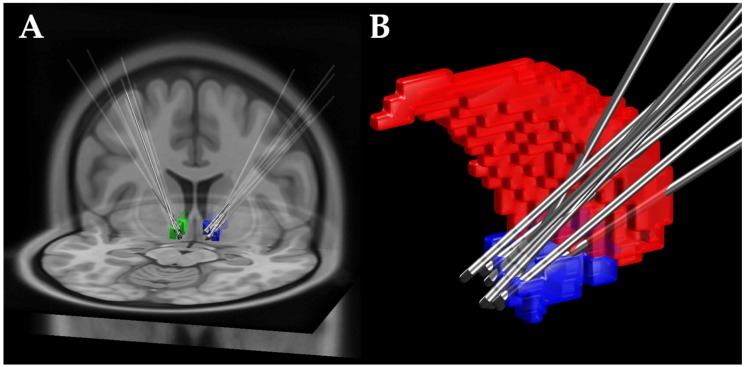
DBS electrode locations: (**A**) The left NAc is depicted in green and the right NAc in blue. (**B**) In the close up view the NAc is depicted in blue and the nucleus caudatus in red. (DBS, deep brain stimulation; NAc, nucleus accumbens).

All severely affected patients with SUD received NAc-DBS as part of the NASA or DeBraSTRA study, respectively. For visualization of the DBS electrodes in respect to the NAc we used the lead-DBS toolbox [58]. Electrode coordinates were calculated using a preoperative Magnetic Resonance Imaging and a postoperative computed tomography (see Figure 2).

### 2.3. Questionnaires

In the scope of the present investigation, all participants filled out a demographic questionnaire with information on age, handedness, years of education and current drug or alcohol use. Depression symptoms were administered with the Beck Depression Inventory (BDI; score ≥ 15 has clinical relevance) in all subjects [59].

### 2.4. Delay Discounting Task

#### 2.4.1. Behavioral Pretest

Subjects performed a short adaptive pretest to estimate the individual discounting rate, which was then used to generate subject-specific trials for the subsequent testing session (see [60]). We used a hyperbolic function where SV is the subjective (discounted) value, D the delay in days and k the individual choice behavior.

(1) $$\mathrm{SV}=\frac{1}{1+kD}$$

Individual choice behavior is characterized by an individually fitted k-parameter. The hyperbolic model is one of the most frequently used models [61]. Equation (1) was used to create subject-specific trials with Matlab (The MathWorks, Inc., Natick, MA, USA) (see Peters and Büchel, 2009 [60]). All experiments were programmed in Presentation (Presentation software; Neurobehavioral Systems, Inc., Berkeley, CA, USA).

#### 2.4.2. Experimental Session

We used a classical DD task in which participants were repeatedly (140 times) presented with two options: either receiving 20 € immediately (immediate option), or being paid varying, uniformly spaced amounts (min. 20.5 €, max. 80 €) after a delay (1, 2, 7, 14, 30, 90 or 180 days) (delayed option). Participants were informed that after the experiment one trial would be selected at random and that their actual choice would determine the amount and the delay of the payout. They were paid immediately in cash when an immediate trial was selected or the money was transferred to their banking account after the respective delay when a delayed trial was selected.

**Figure 3:**
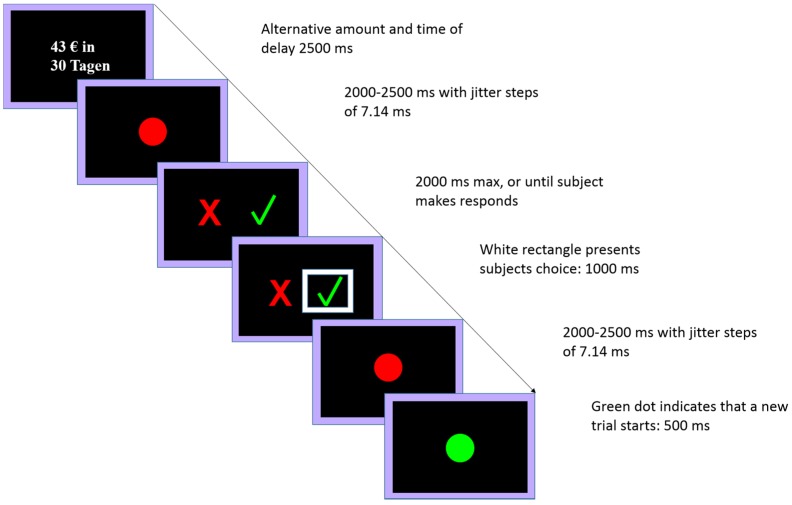
Depiction of one trial, subjects must choose between 20 € now (default option) and a larger but delayed reward.

The screen displayed the alternative amount only and the time delay (white on black background) for 2500 ms. Then, a red dot was presented on the screen for a maximum of 2000 ms or until the subject pressed a button. Participants chose the cross or the tick to refuse or accept the alternative offer, respectively. After they responded, within 200 ms, the chosen symbol (cross or tick) was marked with a white rectangle for 1000 ms, and a new trial started with a red dot for 2000 to 2500 ms (m = 2250 ms with jitter steps of 7.14 ms) that turned green for a 500 ms, indicating the start of a new trial (see Figure 3). In case that responses exceeded 2000 ms, the instruction “Please respond faster!” was presented instead of the red dot. 

### 2.5. Computational Modeling

Following standard procedures (see Peters and Büchel [62]) the probability of choosing the selected option on a given trials was modeled using softmax action selection with Equation (2):

(2) $$P\left(\mathrm{chosen}\right)=\frac{\exp\left({\mathrm{SV}}_{\mathrm{chosen}}/\mathrm{temp}\right)}{\exp\left({\mathrm{SV}}_{\mathrm{other}}/\mathrm{temp}\right)+\exp\left({\mathrm{SV}}_{\mathrm{chosen}}/\mathrm{temp}\right)}$$

Here, temp is a free parameter that models stochasticity in the choices under the given model. SV (subjective value) for smaller sooner rewards was simply the nominal amount (20 €). For later larger rewards, SV was calculated according to the hyperbolic model with Equation (3):

(3) $$\mathrm{SV}=\frac{A}{\left(1+k*D\right)}$$

Here, k is a subject specific discount rate, were larger values correspond to steeper devaluation of rewards over time. A is the nominal amount of the larger later reward (in Euros) and D is the delay to the larger later reward (in days).

Modeling was carried out using two analyses. First, using optimization procedures implemented in Matlab, we fitted individual subject choice data using Maximum Likelihood estimation (fminsearch), obtaining point estimates for each participant, session (NAc-DBS on vs. DBS off) and model parameter separately.

Second, we used a hierarchical Bayesian estimation approach fitting all data of all participants and sessions using Markov Chain Monte Carlo (MCMC) sampling via Just Another Gibbs Sampler (JAGS) [63]. Individual choice data were modeled using Equations 2 and 3 (see above). However, single subject parameters were drawn from group-level normal hyper-distributions, with mean and variance hyper-parameters that were themselves estimated. We assumed four separate group level distributions (patients: DBS on, DBS off; controls: session #1, session #2) with broad uninformative prior distributions defined over sensible intervals. That is, for the means of the group-level hyper distributions of k, uniform priors over the interval [0.00001, 2] were used. For temp, the priors for the mean of the group level hyper-distributions were set to normal distributions with mean 20 and variance 1000 truncated at 0 (i.e., essentially flat priors).

MCMC sampling proceeded via two separate chains with a burn-in of 2000 samples, thinning of 2 and a final number of 20.000 retained samples. The posterior distributions of the group-levels hyper-parameters were then compared between groups and DBS conditions to examine the effects of DBS on discounting behavior (k) and decision noise (temp).

### 2.6. Model-Free Analysis

**Figure 4:**
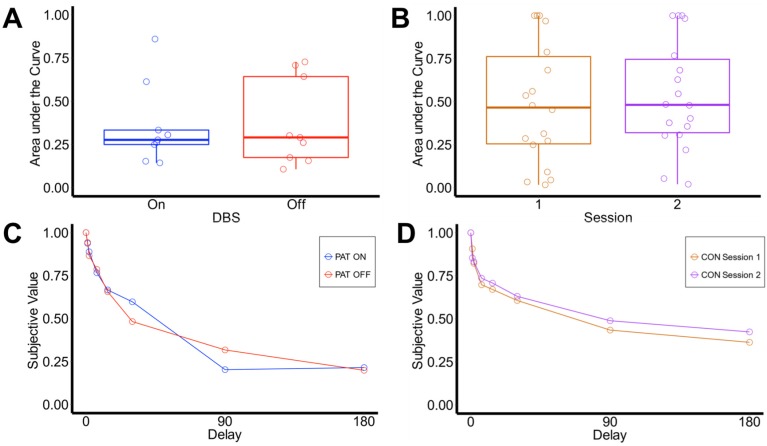
(**A**) Boxplots for AUC-values of NAc-DBS patients with SUD and (**B**) healthy controls; (**C**) depiction of subjective value as a proportion of objective value for NAc-DBS patients with SUD and (**D**) healthy controls. (CON, Healthy controls; DBS, Deep brain stimulation; Pat, Patients with SUD).

In contrast to our computational model based analysis of discounting behavior, we also used an established model-free approach. A model-free estimation of discounting behavior can avoid some problems associated with model-based analysis, e.g., problems with parameter estimation, the choice for a theoretical framework (hyperbolic or exponential models) or extreme parameter estimates that result in skewed distributions. This might yield problems for statistical approaches that require normally distributed variables. We therefore supplemented the model-based analysis using the area under the empirical discounting function (AUC) [64]. In detail, the AUC corresponds to the area under the connected data points that describe the decrease of the subjective value (y-axis) over time (delay; x axis) (see Figure 4C for patients with SUD and 4D for healthy controls). Each specific delay was expressed as a proportion of the maximum delay [64] and plotted against the normalized subjective (discounted) value. We then computed the area of the resulting trapezoids using the following Equation (4):

(4) $$\frac{x_{2}-x_{1}}{\left(\frac{\left(y_{1}+y_{2}\right)}{2}\right)}$$

The sum of all trapezoids then reflects the individual AUC. Higher AUC-values reflect less discounting and lower AUC values reflect more discounting (range from 0 to 1). The analysis was conducted with SPSS version 25.0 (IBM corporation, Armonk, New York, NY, USA) and customized Matlab routines. We applied a paired *t*-test to test a possible stimulation effect in patients with SUD (DBS on vs. DBS off). To compare DBS on and DBS on vs. session #1 and session #2, we applied independent *t*-tests. Problematic here is, that around half of the patients with SUD first had the DBS on session and the other half first the DBS off session. To address this potential distortion we also averaged for each patient DBS on and DBS off and for each healthy control session #1 and session #2. Then we used the averaged transformed *AUC*-values to perform *t*-tests for independent groups.

Moreover, we examined the group-level posterior distributions for log(k) and temp that were estimated using MCMC via the hierarchical Bayesian model. 

Furthermore, we compared age, years of education and BDI scores with the Mann Whitney-U test and gender and handedness with the *Χ*^2^-test.

## 3. Results 

### 3.1. Demographic Characteristics 

We found no significant group effects for age (*p* = 0.860), gender (*p* = 0.149), handedness (*p* = 0.667) or BDI score (*p* = 0.106). Patients with SUD differed in their years of education compared to healthy control subjects (*p* = 0.001). Demographic characteristics and BDI scores of patients with SUD and NAc-DBS and healthy controls are shown in Table 2.

### 3.2. Delay Discounting

#### 3.2.1. Area under the Curve

Patients with SUD did not differ between DBS on and DBS off (*t*(8) = −0.305, *p* = 0.768)). Patients with SUD and healthy controls did not differ in their AUC-values during DBS on and session 1# (*t*(25) = −1.162, *p* = 0.257) and DBS off and session 2# (*t*(25) = −1.314, *p* = 0.168). To account for a possible distortion due to the counterbalanced design in patients with SUD, we also compared averaged DBS on/DBS off and session 1#/session 2# scores. Patients with SUD and healthy controls also did not differ in their averaged AUC-values (*t*(25) = −1.367, *p* = 0.185) (see Figure 4A,B). 

#### 3.2.2. Computational Modeling

**Figure 5:**
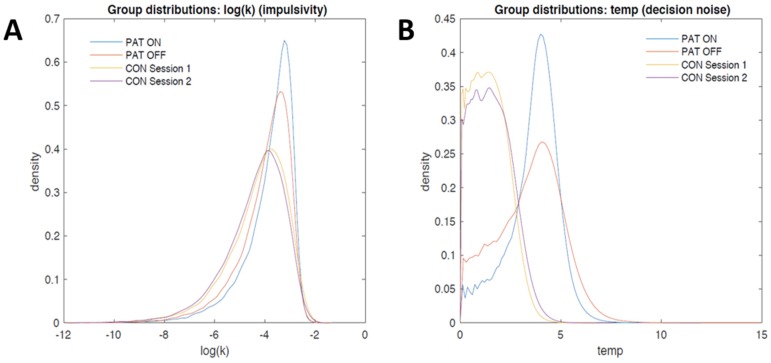
(**A**) Group-level posterior distributions of log(k) for patients and controls; (**B**) Group-level distributions of decision noise (temp) for patients with SUD and controls. (CON, Healthy controls; Pat, Patients with SUD).

We next examined the posterior distributions of the group-level parameter distributions for log(k) and temp (see Methods Section), which are plotted in Figure 5. Although numerically, the mean of log(k) tended to be higher for the SUD group (Figure 5A), there was substantial overlap between groups as well as between the distributions of DBS on vs. off. Likewise, although the mean of the group level temp distribution was somewhat higher for the SUD group (Figure 5B), there was still substantial overlap in the distributions between groups as well as between DBS on and DBS off.

**Figure 6:**
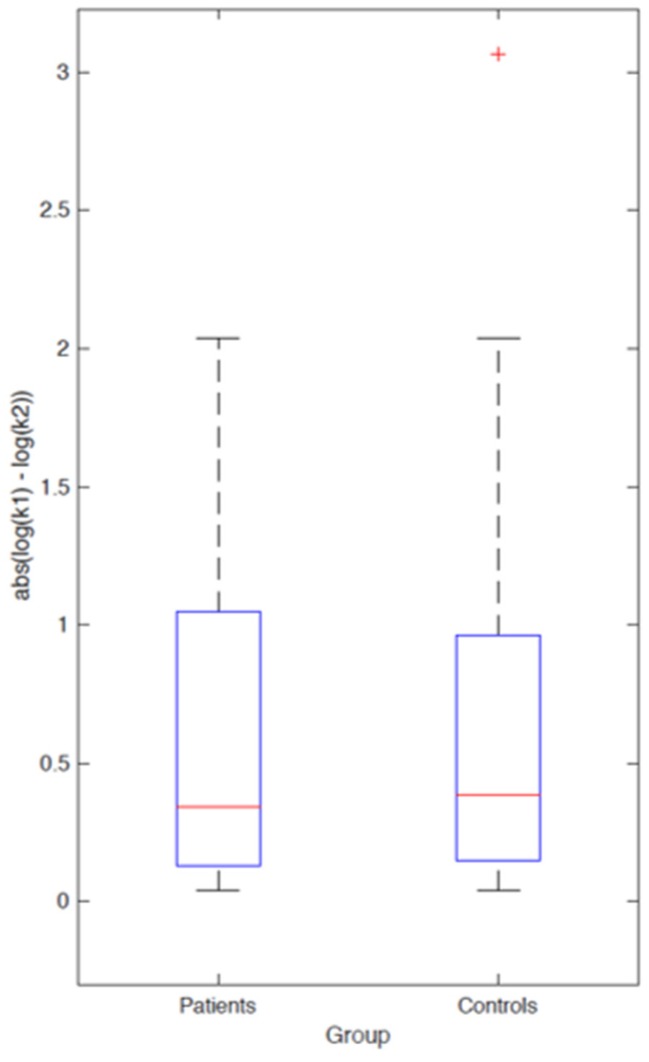
Absolute differences in log(k)-values between testing sessions in patients with SUD and healthy controls.

One potential problem with such an analysis approach is that it merely tests for effects of consistent directionality between groups. We therefore explored the possibility that DBS might exacerbate absolute differences in model parameters, regardless of the directionality of the effects. Figure 6 therefore plots $\left|\log\left(k_{session1}\right)-\log\left(k_{session2}\right)\right|$ for both groups using the Maximum Likelihood parameters. The boxplots again highlight essentially complete overlap in the parameter distributions, which argues against the idea that DBS might increase *k*-values in some of the patients and decrease them in others, since such an effect would increase absolute parameter differences between sessions in patients with SUD vs. healthy controls.

**Figure 7:**
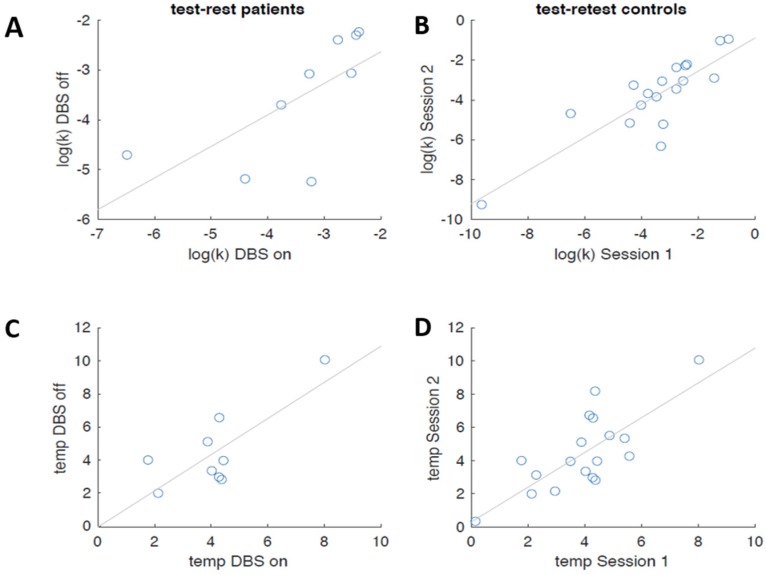
Retest stability (log(k) value) in (**A**) DBS patients with SUD and in (**B**) healthy controls. Decision noise (temp) in (**C**) DBS patients with SUD and in (**D**) healthy controls (DBS, deep brain stimulation; SUD, substance use disorder).

In the light of the surprising absence of group differences with respect to k (see above), we next checked the test-retest reliabilities of our measures in each group. The estimated log(k) parameters between sessions were correlated with *r* = 0.613 (*p* = 0.001) for patients with SUD and with *r* = 0.678 (*p* = 0.045) for healthy controls. The estimated temp parameters (decision noise) between sessions were correlated with *r* = 0.731 (*p* = 0.025) for patients with SUD and 0.928 (*p* < 0.001) for healthy controls (see Figure 7). These analyses confirm robust stability of discounting behavior (see [65]) also in the groups tested here.

## 4. Discussion and Conclusions

To our knowledge, this is the first study elucidating the association of NAc-DBS in patients with severe SUD and the ability to delay rewards. DD is a well-known paradigm with reliable associations to SUD [20] and a high retest-stability [65]. DD directly scales with treatment outcomes [66] and the severity of drug abuse [9]. Therefore, we decided to use DD to assess one important aspect of self-control in patients with severe SUD that were treated with NAc-DBS.

Contrary to our first hypothesis, our findings indicate that a 24 h washout period of NAc-DBS does not alter discounting behavior in patients with SUD. Contrary to our second hypothesis, patients with SUD do not differ in their discounting behavior compared to healthy controls. A number of complementary analytical approaches support our DD findings. First, results were similar for model-based and model-free (AUC) estimation of discounting behavior. This suggests that the absence of behavioral effects is unlikely attributable to particular modeling decisions such as the focus on the hyperbolic model. The robust stability of discounting behavior is also reflected in the high retest stability in patients with SUD and healthy controls. Second, to address the possibility that DBS may have had effects with a different sign in different patients with SUD, we also compared the absolute difference in model parameters between groups. This revealed that DBS-related effects in patients with SUD had a magnitude similar to variance between testing sessions in controls, arguing against the idea that DBS might make some patients with SUD show more self-control and others less. Finally, we complemented the maximum-likelihood-based assessment of individual subject point estimates with a hierarchical Bayesian approach. However, the latter also revealed considerable overlap between the group distributions of both temp and log(k).

Regarding the absent statistical difference between on and off NAc-DBS our results are in line with the results of DD in patients with Parkinson’s disease with DBS of the subthalamic nucleus [67,68]. The absent statistical significant difference between patients with SUD and healthy controls is in contrast to previous findings that indicate robustly increased discounting in clinical samples of patients with SUD [9,20]. Since the patients in this study were severely affected by their respective disorders we would have expected to replicate those findings. A possible explanation for our findings might be that continuous NAc-DBS indeed decreased discounting behavior in patients with SUD, but this effect was not sufficiently interrupted by a 24 h washout period. Importantly, several limitations have to be considered regarding this speculative explanation. First, we were unable to assess DD prior to DBS implantation because the DD task was not included in the initial clinical studies. In addition, the DD assessment was planned after the majority of patients with SUD were already included in the clinical studies, which precluded baseline measurements. Based on our results we strongly encourage baseline assessment in future studies to disentangle stimulation effects more unequivocally. Second, the 24 h washout period was chosen based on previous findings in patients with obsessive compulsive-disorder (OCD) treated with NAc-DBS [69,70,71,72] and considerations regarding the patient’s wellbeing (i.e., avoidance of intense craving or relapse). Importantly, DBS in a certain target might have vastly different washout periods in different disorders such as in Parkinson’s disease (minutes) and dystonia (weeks) [73]. We suggest that future studies might examine different washout periods, but also want to emphasize patient safety in this regard. Third, a potential caveat of the study is the small sample size, which results in small statistical power. Due to the novelty of the therapeutic approach, and therefore lack of available data on effect sizes regarding continuous NAc-DBS or the 24 h washout on DD, we were unable to perform a-priori power analysis. We acknowledge that an effect might have been missed due to the small sample size of our study and strongly encourage further investigation. Underpowered statistics have always been unpopular, but are often unavoidable in clinical samples. Therefore, with respect to the experimental and innovative character of NAc-DBS in patients with SUD, we nonetheless believe that the present study—with, to our knowledge, the largest sample of patients with SUD treated with NAc-DBS—has considerable value to inform an emerging field [74]. Fourth, the patients in our sample had a significantly lower education than the healthy controls. Lower education has been linked with increased discounting behavior, which might have influenced the results of this study [75]. Since no significant effects were observed, this influence might be negligible. Fifth, patients with different SUDs were included in this study, which might have further influenced our results. This limitation might be partially mitigated by the fact that the absolute differences in model parameters was not significantly altered, arguing against a large within-group difference.

In summary, this study indicates that short-term discontinuation of NAc-DBS did not extensively alter self-control operationalized as DD. In addition, patients with SUD that are treated with NAc-DBS did not differ significantly in their discounting behavior from healthy controls. One potential interpretation of these results might be that long-term NAc-DBS indeed changes DD but the 24 h washout period is insufficient to alter this long-term effect. However, since assessments of baseline DD are lacking, this interpretation has to be regarded as speculative. The alternative explanation that NAc-DBS has no or a minor effect on DD cannot be dismissed. Furthermore, the lack of statistical power due to the small sample size might have concealed a difference between healthy controls and patients with SUD. Future studies might expand on our findings and should also address other aspects of self-control like motor impulsivity [76], cognitive control [77] or episodic future thinking [78].

## Figures and Tables

**Table 1:** Overview of disorder, sex, age, duration of addiction before surgery and stimulation parameters (monopolar, case anode, all bilateral) of DBS patients with substance use disorder (SUD).

ID	Disorder	Sex	Age	Years of Addiction	Electrode Contacts	Frequency	Amplitude	Pulse-Width
(1)	Opioid	M	53	35	−0, −1	130 Hz	3.5 V	90 µs
(2)	Opioid	M	58	40	−0, −1	130 Hz	3.5 V	90 µs
(3)	Opioid	M	47	24	−0, −1	130 Hz	3.5 V	90 µs
(4)	Opioid	M	24	4	−0, −1	130 Hz	2.7 V	90 µs
(5)	Opioid	F	34	7	−0, −1, −2	130 Hz	2.5 V	90 µs
(6)	Alcohol	M	47	31	−0, −1	130 Hz	4.5 V	90 µs
(7)	Alcohol	M	38	6	−2, −3,	130 Hz	L: 6 V R: 5.5 V	L: 450 µs R: 240 µs
(8)	Alcohol	M	31	13	−2, −3	130 Hz	1.5 V	90 µs
(9)	Alcohol	M	51	30	−3	130 Hz	3.5 V	210 µs

DBS, deep brain stimulation; F, Female; Hz, Hertz; L, left; M, Male; µs, Microsec; R, right; SUD, substance use disorder; V, Volt.

**Table 2:** Demographic characteristics of NAc-DBS patients with SUD and healthy controls.

	Patients with SUD (*n* = 9)		Healthy Controls (*n* = 18)		*U/Χ^2^*	*p*
	Mean	SD	Mean	SD		
Age (Years) ^a^	42.6	11.3	43.4	10.6	77.6	0.860
Years of education ^a^	10.6	1.1	12.3	1.2	28.5	0.001
Male ^b^	88.9%	-	61.1%	-	2.2	0.149
Right-handed ^b^	100%	-	94.5%	-	0.5	0.667
Disease duration	21.1	13.8	-	-	-	-
BDI ^a^	15.0	15.7	5.1	6.2	49.5	0.106

BDI, Becks depression inventory; DBS, Deep brain stimulation; NAc, Nucleus accumbens; SUD, Substance use disorder; ^a^ Mann–Whitney-*U*-test was used because data was not normally distributed, ^b^ *Χ*^2^ square test.
